# Effects of lifestyle intervention in persons at risk for type 2 diabetes mellitus - results from a randomised, controlled trial

**DOI:** 10.1186/1471-2458-11-893

**Published:** 2011-11-25

**Authors:** Vegard Nilsen, Per S Bakke, Frode Gallefoss

**Affiliations:** 1 Department of Internal Medicine, Sorlandet Hospital Kristiansand, Norway; 2 Institute of Internal Medicine, University of Bergen, Norway; 3 Department of Pulmonary Medicine, Sorlandet Hospital Kristiansand, Norway

**Keywords:** type 2 diabetes mellitus, prevention, lifestyle, obesity

## Abstract

**Background:**

Lifestyle change is probably the most important single action to prevent type 2 diabetes mellitus. The purpose of this study was to assess the effects of a low-intensity individual lifestyle intervention by a physician and compare this to the same physician intervention combined with an interdisciplinary, group-based approach in a real-life setting.

**Methods:**

The "Finnish Diabetes Risk score" (FINDRISC) was used by GPs to identify individuals at high risk. A randomised, controlled design and an 18 month follow-up was used to assess the effect of individual lifestyle counselling by a physician (individual physician group, (IG)) every six months, with emphasis on diet and exercise, and compare this to the same individual lifestyle counselling combined with a group-based interdisciplinary program (individual and interdisciplinary group, (IIG)) provided over 16 weeks. Primary outcomes were changes in lifestyle indicated by weight reduction ≥ 5%, improvement in exercise capacity as assessed by VO_2 _max and diet improvements according to the Smart Diet Score (SDS).

**Results:**

213 participants (104 in the IG and 109 in the IIG group, 50% women), with a mean age of 46 and mean body mass index 37, were included (inclusion rate > 91%) of whom 182 returned at follow-up (drop-out rate 15%). There were no significant differences in changes in lifestyle behaviours between the two groups. At baseline 57% (IG) and 53% (IIG) of participants had poor aerobic capacity and after intervention 35% and 33%, respectively, improved their aerobic capacity at least one metabolic equivalent. Unhealthy diets according to SDS were common in both groups at baseline, 61% (IG) and 60% (IIG), but uncommon at follow-up, 17% and 10%, respectively. At least 5% weight loss was achieved by 35% (IG) and 28% (IIG). In the combined IG and IIG group, at least one primary outcome was achieved by 93% while all primary outcomes were achieved by 6%. Most successful was the 78% reduction in the proportion of participants with unhealthy diet (almost 50% absolute reduction).

**Conclusion:**

It is possible to achieve important lifestyle changes in persons at risk for type 2 diabetes with modest clinical efforts. Group intervention yields no additional effects. The design of the study, with high inclusion and low dropout rates, should make the results applicable to ordinary clinical settings.

**Trial registration:**

ClinicalTrials.gov: NCT00202748

## Background

The incidence of type 2 diabetes mellitus is increasing worldwide. Both genetic predisposition and behavioural and environmental risk factors are needed to develop type 2 diabetes [1]. Recent epidemiologic research suggests that the increased incidence of type 2 diabetes is largely due to changes in lifestyle factors such as diet and physical activity [2]. Lifestyle modification in high risk individuals has been proven effective in reducing type 2 diabetes [3];[4], more effective than drug treatment [4] and with sustained reduction in diabetes incidence [5,6]. Cochrane reviews summarizes that exercise combined with diet can decrease the incidence of type 2 diabetes in high risk individuals, but that additional research is needed to reveal the best type of diet [7,8]. According to the International Diabetes Federation, up to 80% of type 2 diabetes is preventable by adopting a healthy diet and increasing physical activity. Even small weight losses combined with about 30 minutes of activity per day, are in many instances enough to prevent or at least postpone the disease [3,4]. One kg of weight lost is associated with a 16% reduction in diabetes risk [9].

Meta-analysis indicate that dietary counselling interventions for persons with obesity or overweight produce modest weight losses that diminish over time [10]. Compared with diet alone, diet in combination with exercise gives a 20% greater initial and sustained weight loss after one year [11]. Successful weight loss studies are usually conducted in tightly randomised, controlled trials (RCTs) with low inclusion rates and low external validity and applicability to clinical practice (Efficacy studies; "Can it work?") [12]. Effectiveness studies ("Does it work?) are usually studies with looser study designs (often simple audits or before-after designs), high inclusion rates, and brief feasible interventions, with focus on the ability to maintain the intervention as standard practice [12]. Patients included in such studies are more often in alignment with patients met in common clinical settings. There is an unmet need to develop practical, sustainable and low-intensity interventions for the large number of people at risk for type 2 diabetes [13]. In this trial, individual lifestyle counselling by a physician, with emphasis on diet and exercise, was provided for individuals at risk of type 2 diabetes. The effects of this intervention, alone or combined with an additional group-based interdisciplinary program over 16 weeks, was assessed in a randomised, controlled design with an 18 month follow-up.

## Methods

### Subjects and study design

The "Finnish Diabetes Risk score" (FINDRISC) was used to identify individuals at high risk for type 2 diabetes, assessing waist circumference, body mass index (BMI), age, medication against high blood pressure, activity, history of high blood glucose and daily consumption of vegetables/fruits. FINDRISC is found to be a simple and feasible tool, i.e. fast, non-invasive, reliable and at the start of this trial, the best available tool for use in clinical practice (14;15). It is also a good predictor of coronary artery disease (CAD), stroke and total mortality [16]. The total score ranges between 0-20. A FINDRISC-score ≥ 9 is found to identify > 70% of new cases of drug treated type 2 diabetes within five years [14]. Hence, all general practitioners (GPs) in the four nearest municipalities to the hospital were each supplied with ten FINDRISC-questionnaires by post, asked to use them on patients at risk for type 2 diabetes. They were requested to refer individuals aged 18-64 with a FINDRISC-score ≥ 9 to the hospital. The Regional Committee for Medical Research Ethics of southern Norway approved the study.

All referred individuals were assessed by the same physician in a clinical examination. A thorough conversation about family history of diabetes and heart disease was carried out, as well as tobacco and alcohol consumption assessments. Finally, the following information, statements and advices were given:

1. the probability of type 2 diabetes can be reduced by 50% with only small changes in lifestyle and weight

2. the same changes can reduce the probability for heart disease considerably

3. The following were emphasized:

• to increase the consumption of fruit and vegetables

• to get at least 30 minutes of activity pr. day

• to achieve at least 5% loss of weight

• to reduce the consumption of sugar and saturated fat

• to use oil as the main source of fat

• to consume cod-liver oil daily

**Figure 1:**
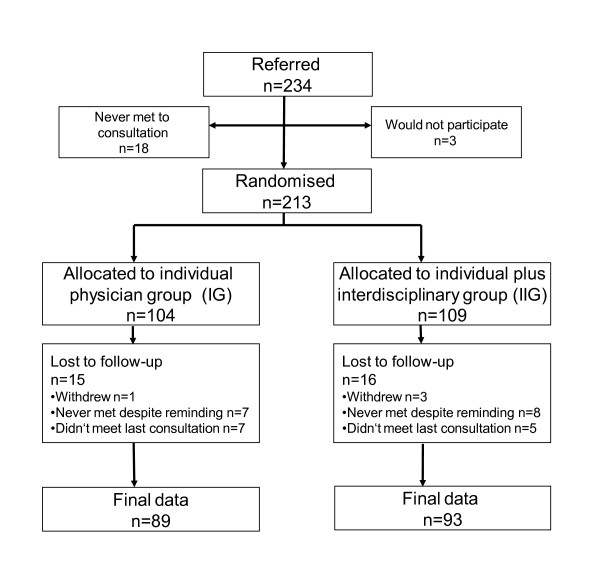
**Flow of participants through trial**.

At the end of the consultation, participants were asked if they wanted to participate in the study. Exclusion criteria were: a diagnosis of diabetes mellitus, the presence of serious heart, lung, kidney or liver failure, serious psychiatric illness, substance abuse and not mastering the Norwegian language. A written informed consent was signed. They were randomly assigned to an "individual physician group" (IG) or an "individual plus interdisciplinary group" (IIG) by use of closed envelope method with unknown block sizes. All GPs received written information about inclusion, group allocation and aims and advices given. Flow of participants through the trial is shown in Figure 1.

Participants in the IG group consulted the study physician at six, twelve and eighteen months after randomisation and otherwise received care from their GP as usual. The study physician used elements of motivational interviewing during these consultations.

**Figure 2:**
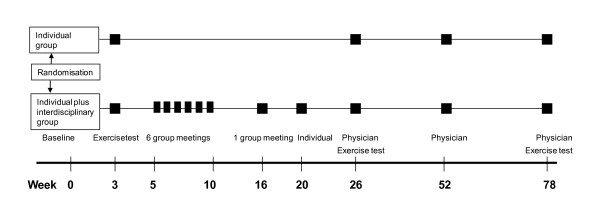
**Overview of the study design**.

In addition, the IIG group participated in a group-based program (≤ ten participants), one day (five hours per day) each week for six weeks and a new gathering after twelve weeks. A systematic review of their situation was given, with emphasis on how to avoid diabetes and CAD, by increasing the level of knowledge and self-consciousness (Figure 2). The topics for these group sessions were research findings and factual information about nutrition and physical activity, habit change, action plans, risk situations, coping strategies, etc. The group intervention also included a variety of physical training. The IIG program was interdisciplinary (dietician, physiotherapist, ergonomist, nurse and physician). Motivational interviewing techniques were utilised. This is a well-known, scientifically-tested method-, which outperforms traditional advice given in the treatment of a broad range of behavioural problems and diseases [17]. An individual 30-minutes consultation with a nurse or ergonomist completed the intervention one month after the last group meeting.

### Assessments

At every visit to the study physician, the following assessments were performed: fasting blood sample, systolic and diastolic blood pressure (SBP and DBP) according to recommended standards [18], waist circumference at a level midway between the lowest rib and the iliac crest to the nearest cm, height without shoes to the nearest cm (only first visit) and weight in indoor clothes to the nearest 100 g. Blood pressures were measured by an Omron M41 and weight with a Seca 771. An oral glucose tolerance test (OGTT), required to rule out diabetes and to identify patients with impaired glucose tolerance (IGT), was not performed prior to nor during the study. These pragmatic inclusion criteria fits well with the aim of the study to test the effects of step one life style intervention in a group at risk for diabetes. The Smart Diet Score questionnaire was used; a fast, simple and validated tool for food assessment resulting in a diet score which ranges between 15 and 45 points [19]. A diet score between 15-29 points is categorised as "unhealthy", 30-37 points as "somewhat unhealthy" and ≥ 38 points as a "healthy" diet. A question was added to the questionnaire to ascertain the number of days with cod-liver oil consumption during the last week.

A physical test on a treadmill was carried out during the first month after randomisation and repeated after six and eighteen months, to determine maximal aerobic capacity (VO2max), utilising a modified Bruce protocol designed for people in poor physical condition [20]. The results were categorised into six levels according to normative data for VO2max for gender and age: very poor, poor, fair, good, excellent and superior aerobic capacity [21]. An increase in exercise capacity of 3,5 ml/kg per minute (one metabolic equivalent (MET)) is shown to be associated with a 12 percent improvement in survival [22].

### Definition of end points

Primary outcomes were changes in lifestyle according to established goals that have been shown to reduce incidence of type 2 diabetes, improve health and to improve cardiovascular risk profile. These were defined as:

• weight reduction ≥ 5% [23]

• reduction in waist circumference of ≥ 5 cm [24]

• improvement in exercise capacity of one MET [22]

• consumption of cod-liver oil ≥ five days per week [25]

• ≥ 4 point increase in Smart Diet Score. The outcome for this diet change is an arbitrary threshold which is not evidence based. It reflects an improvement in four out of 15 areas of diet

### Statistical analyses

Sample size was based upon a decision that a difference between groups in all main outcomes of > 20% was clinically important. Therefore, number needed to treat (NNT) = five, to experience one extra person with a favourable main outcome with the additional group session approach. The spontaneous rate of achieving the primary outcomes was estimated to be approximately 20%. The dropout rate was estimated to 15-20%. On the basis of these assumptions, with a power > 80% (β ≤ 0.20), a significance level α ≤ 0.05, and a two-sided test, the appropriate study size was calculated to be 200 participants, with 100 in each group. Statistical package for Social Sciences 16 (SPSS Inc. Chicago, USA) was employed for statistical analyses. The χ^2 ^test was used to assess the differences between groups when variables were categorical and McNemar test when testing within-group changes from baseline to follow-up. The independent sample t-test was used to assess differences in means between study groups for continuous variables with normal distribution. Paired t-test was used for (within-group) comparisons of quantitative data between baseline and follow-up at 18 months.

## Results

65 GPs out of about 90 referred 234 individuals from March 2004 to September 2005. 216 turned up for consultation (Figure 1). 213 participants were randomised (inclusion rate > 91%) of whom 182 completed the study (> 85%). Mean (standard deviation = SD) FINDRISC score was 12,0 (2,7) for the IG-group and 12,3 (2,8) for the IIG-group. 173 answered the diet questionnaire at the end of study (95% of completers). 201 performed the treadmill test at baseline (94% of included), 168 after six months and 131 (72% of completers) at the end of the study. The dropout rate from baseline to end of study was comparable in the IG- and the IIG group (15%), and comparable between genders. The drop-outs, as compared with completers, were 3,8 years younger (43,2 versus 47,0), more often on antidepressants (23% versus 6%), had higher BMI (38,9 versus 36,4), lower aerobic capacity (24,1 versus 27,2), lower diet score (27,5 versus 29,0) and doubled frequency of both daily smoking (50% versus 21%) and long term sick leave or disability (57% versus 28%), (all p values < 0.05). Participants in the IIG group attended on average five (5,2) of the seven group meetings, and 94% attended the final, individual consultation and assessment.

Randomisation seemed successful for all baseline variables except for BMI. Participants in the IG group had significantly lower BMI than persons in the IIG group (Table 1). 90% of participants were obese (BMI > 30). Weight reducing drugs (orlistat or sibutramin) were used by 10% in the IG-group and 5% in the IIG-group at baseline (p = 0,15), at follow-up they were used by 4% in the IG-group and by 5% in the IIG-group (p = 0,79). None were using metformin or glitazones. Anti-hypertensive drugs were used among 36% of all at baseline and 37% at follow-up. The percentage of subjects with hypertension (defined by systolic blood pressure ≥ 140 mmHg and/or diastolic pressure ≥ 90 mmHg [26] or use of anti-hypertensive drugs) was 71% in the IG-group and 76% in the IIG-group (p = 0,25) at baseline, and 79% and 82% (p = 0,40), respectively, at follow-up. Hypertension were seen more often among subjects using anti-hypertensive drugs compared to subject not using it at baseline, i.e. 75% versus 59%, respectively (p = 0,02), but at follow-up this difference was not significant, 76% versus 65% respectively (p = 0,13).

**Table 1:** Baseline characteristics of 213 included subjects and changes in selected clinical and metabolic variables from baseline to follow-up at 18 months among 182 completers of the study.

	Individual physician group (IG) n = 104			Individual plus interdisciplinary group (IIG) n = 109			All n = 213			
	**Baseline**			**Baseline**			**Baseline**			

**Age**	45,9 (11)			47,0 (11)			46,5 (11)			

**Gender, men, %**	53			47			50			

**Married or cohabiting, %**	79			69			74			

**High school or university, %**	27			29			28			

**Employed, %**	64			61			62			

**BMI**	35,9 (6)			37,6 (6)			36,8 (6)			

	Baseline	Follow-up	P value	Baseline	Follow-up	P value	Baseline	Follow-up	Δ-value	P value

	**n = 89**			**n = 93**			**n = 182**			

**Weight, kg**	111,7 (22)	108,7 (23)	< 0,001*	110,5 (22)	108,0 (20)	0,001*	111,1(22)	108,3 (21)	2,8	< 0,001*

**BMI, kg/m^2^**	35,8 (6)	34,8 (6)	< 0,001*	37,0 (6)	36,2 (6)	< 0,001*	36,4 (6)	35,5 (6)	0,9	< 0,001*

**Waist circumference, cm**	119 (14)	115 (15)	< 0,001*	118 (15)	116 (14)	< 0,001*	118 (14)	115 (14)	3	< 0,001*

**Aerobic capacity, ml/kg/min^2 ^**^2^	27,4 (8)	29,8 (8)	< 0,001*	26,4 (8)	28,7 (7)	< 0,001*	26,9 (8)	29,2 (7)	2,3	< 0,001*

**Heart rate at end of exercise test **^2^	159 (22)	163 (21)	0,009*	159 (19)	161 (21)	0,17*	159 (20)	162 (21)	3	0,004*

**SBP, mmHg**	144 (18)	147 (19)	0,09*	144 (20	143 (19)	0,84*	144 (19)	145 (19)	1	0,37*

**DBP, mmHg**	90 (11)	91 (10)	0,42*	88 (10)	91 (11)	0,03*	89 (11)	91 (11)	2	0,04*

**Fasting plasma glucose, mmol/l**	5,5(0,8)	5,6(0,7)	0,69*	5,6(0,8)	5,8(1,2)	0,06*	5,6(0,8)	5,7(1,0)	0,1	0,08*

**HbA1c, %**	5,6 (0,4)	5,6 (0,5)	0,11*	5,6 (0,4)	5,6 (0,5)	0,91*	5,6 (0,4)	5,6 (0,5)	0	0,29*

**Total cholesterol, mmol/l**	5,5 (1,1)	5,3 (1,0)	0,09*	5,4 (1,1)	5,2 (1,1)	0,07*	5,4 (1,1)	5,3 (1,0)	0,1	0,01*

**HDL cholesterol, mmol/l**	1,18 (0,3)	1,23 (0,3)	0,006*	1,28 (0,4)	1,25 (0,4)	0.17*	1,23 (0,4)	1,24 (0,3)	0,01	0,40*

**Triglycerides, mmol/l**	1,9 (1,0)	1,6 (0,7)	< 0,001*	1,8 (1,4)	1,5 (0,8)	0,01*	1,9 (1,2)	1,5 (0,7)	0,4	< 0,001*

**Diet score, mean**	29 (4)	33 (4)	< 0,001*	29 (4)	34 (3)	< 0,001*	29 (4)	34 (4)	5	< 0,001*

**Healthy diet, % of all**	2	16	0,007#	1	20	< 0,001#	2	18	16	< 0,001#

**Unhealthy diet, % of all**	60	17	< 0,001#	56	10	< 0,001#	58	13	45	< 0,001#

**Daily smoking, %**	21	17	0,22#	18	18	1,0#	20	17	3	0,34#

**Days/week using cod liver oil**	1,8 (3)	3,4 (3)	< 0,001*	1,8 (3)	4,1 (3)	< 0,001*	1,8 (3)	3,7 (3)	1,9	< 0,001*

**Cod liver oil ≥ 5 days per week**	25	43	0,02#	26	54	< 0,001#	25	49	24	< 0,001#

Values are means with standard deviations in parenthesis, unless stated otherwise.

^1^Inter-group differences with p < 0.05 based on Chi-Square test for categorical variables and independent sample t-test for quantitative data

*paired sample t test # McNemar test

^2 ^N = 63 & n = 66 in the IG and IIG group, respectively

Δ-value displays the actual difference between baseline and follow-up

Poor or very poor aerobic capacity was found in 55% of all participants, and was twice as frequent among men (75%) as among women (36%), (p < 0,001). Aerobic capacity at baseline was weakly, inversely correlated with BMI (r^2 ^= 0,22, p < 0,001). An unhealthy diet was found in 60% of all participants, and more frequently among daily smokers (76%) compared with the occasional- and non-smokers (55%), (p = 0,008). More than two-thirds had lower education (primary or secondary education only). For individuals with primary and/or secondary education only, mean diet score was 2,2 points lower (p < 0,001), mean aerobic capacity 4,6 ml/kg/min. lower (p < 0,001) and the frequency of daily smoking more than doubled (30% versus 12%, p = 0,006), compared to those with higher education.

**Figure 3:**
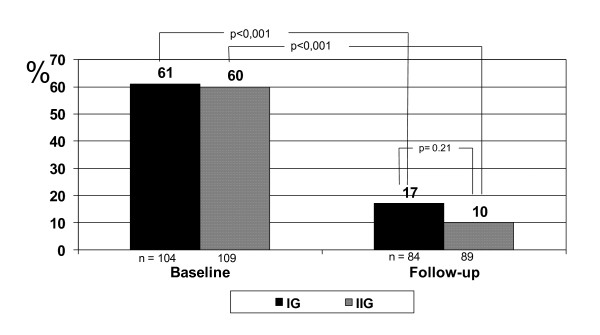
**Reduction in proportion of patients with unhealthy diet from baseline to follow-up**.

From baseline to follow-up there were no significant, additional effects of group intervention (Tables 1 and 2). Thus, the forthcoming results are presented as before-after differences for all participants combined. At least one primary outcome (Table 2) was achieved by 93% while all primary outcomes were achieved by 6%, indicating an important change in lifestyle. Most successful was the 78% reduction in the proportion of participants with unhealthy diet (almost 50% absolute reduction, Figure 3). The number of individuals consuming cod-liver oil ≥ 5 days per week increased by 25% and was thereby doubled. There was a mean increase in maximal aerobic capacity of 9% which was evident after six months and thereafter stable. One third of participants improved their aerobic capacity to an extent which is known to improve health (1 MET). Mean weight loss from baseline was modest: 1,9 kg (SD 5,6), 2,0 kg (SD 6,2) and 2,8 kg (SD 7,1) respectively, at 6, 12 and 18 months assessments, with no gender differences. One-third had a weight reduction ≥ 5% (mean 9,4% (SD 4,0)), one third had a weight reduction less than 5% (mean 2,1% (SD 1,4)) and the last third gained weight (mean 4,0% (SD 3,8)). From baseline to follow-up there were no change in the proportion of participants with plasma glucose ≥ 7,0 mmol/l (6%), IFG (15%) or normoglycemia (79%), and no between group differences.

**Table 2:** Success in achieving primary outcomes by 18 months according to treatment group by proportions (%).

Primary outcome	Individual physician group (IG) n = 89	Individual and interdiciplinary group (IIG) n = 93	P value*	All n = 182
1. Weight reduction ≥ 5%	36	28	0,25	32

2. Waist circumference reduction ≥ 5 cm	42	30	0,11	36

3. Improved diet score ≥ 4 points	55	63	0,28	59

4. Cod-liver oil at least 5 days a week	43	54	0,15	49

Exercise test from baseline to follow-up	**n = 63**	**n = 64**		

1. Improved exercise test ≥ 1MET	35	33	0,80	34

* The χ^2 ^test

## Discussion

This study confirms that changes in lifestyle are possible in individuals at risk for type 2 diabetes, with modest clinical effort. This applies to both genders regardless of educational status. Almost half of participants abandoned their unhealthy diet, one third obtained a health-improving weight loss and one third improved their fitness by one MET. Adding interdisciplinary group-based counselling to the individual physician-based intervention, gave no additional effects.

Limitations of the study must be considered. First, dietary intake was assessed by self-report and may present a source of recall bias. General underreporting compounded with food-specific underreporting is frequent and may increase with increasing BMI [27,28]. Second, 28% of completers failed to perform the treadmill test, which weakens the results for change in fitness. We can consider the worst case scenario i.e. that all who did not attend the last test and all who dropped out did not improve their aerobic capacity. The success rate would then fall from 33 to 20% if success is defined as improvement of VO2max of 1 MET. However, we contend that compliance with treadmill testing for almost three fourths of completers in such an unselected study population is a high standard result. Third, the study-physician (first author) was not blinded to the randomisation status of the participants. This may have biased the results. Fourth, dropouts differed from participants who completed testing by being younger and having poorer lifestyle parameters. Hence, withdrawal in this study does not occur at random, but is more common among individuals who are dissatisfied with their life style [10]. It is a paradox, and a major healthcare challenge, that those who have greatest need for a change in lifestyle are also those who are most likely to discontinue an intervention. Fifth, the generalisability of the findings in this study could be limited by self-selection bias or healthy volunteer bias. Thus, extrapolating these results to the general population may overestimate the effects. However, the results should be valid for patients at risk for diabetes according to the FINDRISC questionnaire.

A major strength of this study is the low drop-out rate compared with other weight loss studies. A meta analysis of 121 pharmaceutical randomised controlled trials with weight loss or weight gain prevention as major end points, found a drop-out rate of 37% at one year [29]. Studies including behaviour modification among overweight and obese out-patients report drop-out rates after one and two years of 53-77% [30,31]. The aim of this study was to evaluate a practical and low-intensity intervention with high external validity. An inclusion rate of > 91% of those referred and a participation rate of > 98% among those who turned up for consultation, no excluded individuals and a drop-out rate < 15%, is in accordance with this aim and increases the general applicability of the study results to common clinical settings. Low education was associated with a poorer diet, lower aerobic capacity and smoking, as found in other studies and reviews [32,33]. These factors and their interactions are possible confounders. These associations were not tested in an interaction term, since such results cannot be utilized in any clinically meaningful way. However, education level did not affect the success with respect to primary outcome achievements.

An unexpected finding was the much higher prevalence of poor or very poor aerobic capacity for gender and age at baseline among males compared with females. Some of the difference can be explained by a lower heart rate among males at the end of the first exercise test. This finding may reflect lower motivation and maximal effort, but may also be influenced by a trend toward more common use of beta blockers among men than women (25% versus 15%, p = 0,08). However, at the final test, both use of beta blockers and maximal heart rate was comparative between genders (20% versus 19%, p = 0,84). Further, the lower aerobic capacity observed in males was not explained by higher BMI. Indeed, BMI in males tended to be lower than in the female group (36,1 versus 37,4, p = 0,10). Therefore given that neither beta blocker use nor BMI differences explain the lower aerobic capacity observed in this group of obese males, we do not have a clear explanation for the difference observed between genders. We note that FINDRISC has a better ability to detect men than women with low aerobic capacity. As far as we know, no one before has previously described the aerobic capacity in individuals screened by FINDRISC.

The short duration and low intensity intervention may explain the absence of additive effect for the group-based, interdisciplinary approach. Svetkey et al found a 8,5 kg initial weight loss in 1032 overweight or obese adults with hypertension/dyslipidemia after six months with 20 group-based meetings, but gradually this weight loss was reduced over the next 30 months to 3,5 kg [34]. Although statistically significant, there was little difference in final weight loss with regard to whether they after the first six months were randomised to monthly personal contact, free use of internet technology or self-directed control. Modest weight loss is nonetheless clinically important since there is a preferential loss of the more pathogenic visceral adipose tissue (VAT) compared with subcutaneous abdominal adipose tissue (SAT) with modest weight loss [35]. A Cochrane review of long-term non-pharmacological weight loss interventions for adults with pre-diabetes, found weight loss of 2,8 kg and 2,6 kg, respectively, after one and two years, which is comparable with the weight loss in this study [36]. Further, the weight loss in this study is even more clinically important if this result is compared with the natural concomitant weight gain found in population-based surveys [37,38].

The effects on glucose metabolism and lipids were modest. Despite the favourable lifestyle changes achieved, no difference was observed in the fasting plasma glucose and HbA1c values, or the proportion of subjects with impaired fasting glucose, within the 18-month study duration. Subgroup analyses including participants with both ≥ 5% weight reduction and improved aerobic capacity ≥ 1 MET (n = 24) showed statistically significant (p < 0.05) changes from baseline to follow-up; a HbA1c reduction from 5,8 to 5,5%, drop in triglyceride levels from 2,0 to 1,3 mmol/l and in total cholesterol from 5,5 to 5,0 mmol/l. Blood pressure was not improved, in fact there was an increase in diastolic blood pressure in the IIG group. The prevalence of hypertension was very high, and higher among users of antihypertensive medications. Subgroup analyses including the same 24 participants from above with both ≥ 5% weight reduction and improved aerobic capacity ≥ 1 MET, showed systolic/diastolic blood pressure reduction of 7/4 mmHg which significantly differed compared to a rise of 3/3 mmHg in the rest of the participants. Favourable metabolic improvements were achieved among subjects who significantly changed their lifestyle, not among the others. Use of anti-hypertensive or lipid lowering drugs did not change during the study.

Is there a lack of knowledge with regard to what persons at risk of type 2 diabetes should do to avoid type-2 diabetes? The "Study to Help Improve Early evaluation and management of risk factors Leading to Diabetes" (SHIELD) demonstrates appropriate knowledge and healthy attitudes in individuals with or at risk for type 2 diabetes [39]. Despite this, only 28% of individuals at high risk for diabetes were exercising regularly and only 14% were following a prescribed diet. Patient empowerment has been advocated as an approach to improve this gap between patient knowledge and behaviour [39], which is comparable to the principles of Motivational Interviewing (MI) used in our study. Although different "dosages" of MI were performed in the IG and IIG groups, both groups were approached with MI, which may partly explain the lack of differences between intervention groups.

Previously published clinical trials show impressive results with relative risk reductions for type 2 diabetes of 58% for individuals with impaired glucose tolerance (IGT) [3,4]. Despite this, the World Health Organization estimates that the number of diabetes deaths will double between 2005 and 2030. In many European countries and in the US, adult obesity has reached epidemic proportions with a prevalence of approximately 34% [38,40], coupled with a 34% prevalence of overweight [38]. Strategies to prevent weight gain on a population level are poorly understood [41] and there remains a lack of evidence for an effective intervention to prevent obesity [42]. To stop the epidemic, collaboration between academic, governmental, industrial and health care sectors is needed [43]. This implies that elements such as food supply, the availability of sweets, transport policy, advertising, labelling and prices have to be evaluated. Until governmental implementation of effective strategies to reduce the invasion of the metabolic syndrome is assured, an individual approach as shown in this study can be utilised with modest clinical efforts and clinically important results.

## Conclusion

FINDRISC identifies subjects with high frequency of unhealthy lifestyle parameters. It is possible to accomplish important lifestyle changes in these subjects with modest efforts to prevent or delay development of type 2 diabetes or cardiovascular disease. Group intervention yields no additional effects. The results should be applicable to ordinary clinical settings.

## Competing interests

The authors declare that they have no competing interests.

## Authors' contributions

In this study all authors participated in the design and coordination of the study. VN conducted literature review, did all the clinical work and is the main author. FG helped to draft the manuscript and provided advice on data analysis. All authors read and approved the final manuscript.

## Pre-publication history

The pre-publication history for this paper can be accessed here:

http://www.biomedcentral.com/1471-2458/11/893/prepub
